# Evidence that a mitochondrial death spiral underlies antagonistic pleiotropy

**DOI:** 10.1111/acel.12579

**Published:** 2017-02-09

**Authors:** Michael Stern

**Affiliations:** ^1^ Department of BioSciences, Program in Biochemistry and Cell BiologyRice UniversityHoustonTXUSA

**Keywords:** aging, Foxo, insulin signaling, mitophagy, reactive oxygen species, Tor

## Abstract

The antagonistic pleiotropy (AP) theory posits that aging occurs because alleles that are detrimental in older organisms are beneficial to growth early in life and thus are maintained in populations. Although genes of the insulin signaling pathway likely participate in AP, the insulin‐regulated cellular correlates of AP have not been identified. The mitochondrial quality control process called mitochondrial autophagy (mitophagy), which is inhibited by insulin signaling, might represent a cellular correlate of AP. In this view, rapidly growing cells are limited by ATP production; these cells thus actively inhibit mitophagy to maximize mitochondrial ATP production and compete successfully for scarce nutrients. This process maximizes early growth and reproduction, but by permitting the persistence of damaged mitochondria with mitochondrial DNA mutations, becomes detrimental in the longer term. I suggest that as mitochondrial ATP output drops, cells respond by further inhibiting mitophagy, leading to a further decrease in ATP output in a classic death spiral. I suggest that this increasing ATP deficit is communicated by progressive increases in mitochondrial ROS generation, which signals inhibition of mitophagy via ROS‐dependent activation of insulin signaling. This hypothesis clarifies a role for ROS in aging, explains why insulin signaling inhibits autophagy, and why cells become progressively more oxidized during aging with increased levels of insulin signaling and decreased levels of autophagy. I suggest that the mitochondrial death spiral is not an error in cell physiology but rather a rational approach to the problem of enabling successful growth and reproduction in a competitive world of scarce nutrients.

**Abbreviations**
AP: antagonistic pleiotropy
IIS: insulin/insulin growth factor signaling
ROS: reactive oxygen species

In the long run we are all dead. Economists set themselves too easy, too useless a task, if in tempestuous seasons they can only tell us, that when the storm is long past, the ocean is flat again.

John Maynard Keynes, 1923

## The death spiral, pros and cons

A death spiral, also known as a vicious circle, is a specific form of positive feedback in which steps taken to handle a particular problem, while successful in the short term, exacerbate the problem in the long term. The classic example of a death spiral is a company with debt trouble that must borrow to pay for operating expenses. Although the operating expenses can get paid (short‐term success), the additional borrowing worsens the company's debt problem (long‐term exacerbation). Insurance companies can face death spirals when, as a consequence of adverse selection, claims increase unexpectedly, which necessitate premium increases, which increase the adverse selection, etc.

The death spiral is generally viewed unfavorably because the end result of a death spiral is generally catastrophic failure. However, in comparison with the alternative, the death spiral offers critical advantages. For example, when a debt‐afflicted company borrows money to pay operating costs, it survives longer – perhaps not forever, but longer at least than it would have in the absence of this activity. Reaping benefits in the long term first requires survival through the short term, as is indicated above in the quote from John Maynard Keynes, and the death spiral can, at least, promote this short‐term survival.

## Aging as a form of death spiral

From an evolutionary perspective, aging has been difficult to understand. Natural selection increases organismal fitness, and yet aging, which clearly decreases fitness, is not only observed, but also appears to be nearly universal within multicellular (and even some single‐celled) organisms. To address this dilemma, it was proposed that aging occurs and is fixed in populations because alleles that have deleterious effects in old age benefit growth, survival, and reproduction in youth. This theory is called antagonistic pleiotropy (AP) theory (Williams, 1957). In this view, aging occurs because alleles that in the short term are beneficial in solving problems in growth and reproduction serve to exacerbate the problem in the long run. Therefore, aging can be viewed as a form of death spiral.

## Evidence that the genes of the insulin signaling (IIS) pathway mediate AP

If this premise is accepted, the next step is to identify the alleles that mediate AP, understand the nature of these alleles, how they might exert AP, and finally identify and define the critical cellular processes affected by AP.

Alleles of genes in the insulin/insulin growth factor signaling (IIS) pathway are the likeliest candidates for AP alleles (Walker *et al*., 2000; Blagosklonny, 2010). Loss‐of‐function alleles in the IIS pathway slow aging and increase lifespan in a variety of invertebrate and vertebrate systems (Kenyon, 2010), which means that wild‐type alleles of the IIS pathway promote aging and decrease lifespan. In *C. elegans*, loss‐of‐function alleles in the insulin receptor *(daf‐2)*,* PI3K (age‐1*) and *Akt* (two redundant genes, in double mutant), the *D. melanogaster* insulin receptor *InR* and insulin receptor substrate *chico*, and the mouse growth hormone‐releasing hormone gene *GHRH* and the insulin growth factor receptor *IGFR1* each delay aging (Clancy *et al*., 2001; Flurkey *et al*., 2002; Holzenberger *et al*., 2003; Kenyon, 2010). Thus, wild‐type alleles of this pathway, by promoting aging and impairing longevity, fulfill the requirement that AP alleles are deleterious to organisms in old age. In addition, members of the Foxo transcription factor family, which are inhibited by IIS, slow aging in a number of systems (Martins *et al*., 2016).

IIS genes also fulfill the requirement that AP alleles promote growth and reproduction at young ages. Loss‐of‐function mutations in IIS genes confer many deleterious effects to young organisms, including very slow growth, dwarfism, and deficient fecundity. It is unlikely that such mutants could reproduce or even survive in the wild. Taken together, these results indicate that wild‐type alleles of the IIS pathway promote growth and reproduction in young organisms at the expense of rapid aging.

## IIS pathway activity increases protein translation and inhibits autophagy

**Figure 1:**
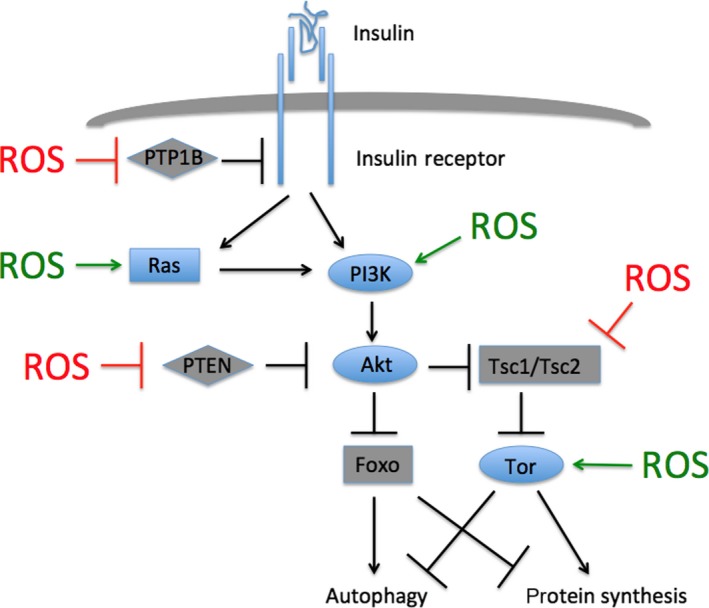
The insulin/insulin growth factor signaling pathway and its activation by reactive oxygen species (ROS). Insulin or other growth factors bind to and activate the insulin receptor or other receptor tyrosine kinases. This binding leads to PI3K and Akt activation either directly or via Ras. Akt phosphorylates and inhibits the activities of Foxo and the Tor inhibitor Tsc1/Tsc2. Activated Tor impairs autophagy and activates protein synthesis, whereas activated Foxo has the opposite effects. Phosphatases inhibit signaling either by catalyzing receptor dephosphorylation or by antagonizing PI3K activity. Pathway activators are shown in blue, and inhibitors in gray. ROS inhibits pathway inhibitors (red) and activates pathway activators (green).

The accelerated aging by IIS pathway activity is most likely mediated by one or more of the cellular outputs regulated by IIS. These outputs include autophagy, which is inhibited by IIS, and cell growth, which is activated by IIS (Kapahi *et al*., 2010). Two molecular targets of IIS that regulate each include the Tor kinase (Schmelzle & Hall, 2000), which is activated by IIS (Hay & Sonenberg, 2004), and the Foxo transcription factor, which is inhibited by IIS (Tang *et al*., 1999; Fig. 1). Both Tor and Foxo have been implicated in mediating the effects of IIS on aging; Foxo activity promotes longevity and Tor activity promotes aging (Kenyon, 2010). Consistent with these observations, Tor and Foxo regulate a strongly overlapping series of outputs, but in opposite directions. Tor inhibits autophagy by directly phosphorylating and inhibiting critical autophagy proteins such as ATG13 (Kamada *et al*., 2010) while simultaneously promoting protein synthesis by phosphorylating and inhibiting the translation inhibitor 4E‐BP (Hay & Sonenberg, 2004; Kim *et al*., 2011; Fig. 1). In contrast, Foxo activates autophagy by activating transcription of autophagy genes *ATG8* and *ATG12* while simultaneously inhibiting protein synthesis by activating *4E‐BP* transcription (Jünger *et al*., 2003; Webb & Brunet, 2014; Fig. 1). These effects of Tor and Foxo on autophagy components are physiologically significant. Tor activation decreases autophagy (Kim *et al*., 2011), whereas loss of Foxo decreases autophagy in muscle and other tissues (Mammucari *et al*., 2007).

Autophagy is likely to be an important process for control of aging (Rubinsztein *et al*., 2011; Tower, 2015). As a quality control mechanism that ensures adequate function of proteins and organelles over time, autophagy would enable cells to maintain viability over long periods. Indeed, inhibiting autophagy confers cellular deficits related to aging (Blagosklonny, 2010; Rubinsztein *et al*., 2011). Furthermore, autophagy declines during normal aging in Drosophila muscle (Demontis & Perrimon, 2010), mouse lung (Shirakabe *et al*., 2016), and human brain (Lipinski *et al*., 2010), and the mitochondrial autophagy (mitophagy) inducer *PINK1* is transcriptionally downregulated during aging in mouse lung (Sosulski *et al*., 2015). It was previously proposed that IIS pathway activity is deleterious to old organisms via inhibition of autophagy (Blagosklonny, 2010; Gems & de la Guardia, 2012).

Although autophagy is responsible for degrading many types of damaged organelles or other macromolecular structures, mitophagy is likely to be the process most critical for aging. First, an early theory of aging posited that cellular damage caused by free radicals or reactive oxygen species (ROS; Harman, 1956) is a major cause of aging. ROS chemically modify a number of different functional groups on proteins, lipid, and DNA and thereby cause dysfunction. Mitochondria are a potent source of ROS generation and therefore would be expected to be particularly susceptible to ROS‐mediated damage. Second, alone among organelles and other macromolecular structures within animal cells, mitochondria possess DNA, which encodes several proteins critical for oxidative phosphorylation. Whereas every other macromolecular structure can be perfectly reconstructed with only nuclear genomic input, mitochondria are uniquely dependent on non‐nuclear DNA for continued activity. Thus, ROS‐mediated mitochondrial DNA damage, if allowed to persist, irreversibly impairs mitochondrial function. With time, the continuous accumulation of mitochondrial DNA mutations would continuously ratchet down mitochondrial ATP productive capacity, and be primarily responsible for the decline in cellular function over time.

## IIS inhibits mitochondrial quality control by inhibiting mitophagy

Cells possess numerous mechanisms to enable maintenance of mitochondrial function and genome integrity over time, despite continuous generation of mitochondrial mutations. Most notably, cells possess mechanisms that enable the detection, segregation, and finally mitophagic destruction of dysfunctional mitochondria, in a process termed mitochondrial quality control. The importance of this quality control in cell physiology is demonstrated by experiments showing that mitophagy inhibition decreases bulk mitochondrial oxidative phosphorylation capacity and causes deficits in cell function (Twig *et al*., 2008).

As described above, IIS inhibits autophagy in general. This autophagy inhibition leads to long‐term declines in mitochondrial health: Long‐term (10‐week) Tor activation in the heart increases mitochondrial number, but decreases mitochondrial output, both phenotypes likely a consequence of impaired mitophagy (Grevengoed *et al*., 2015). Furthermore, IIS specifically inhibits transcription of the mitophagy inducer *PINK1* (*PTEN‐induced Kinase 1*), which was originally identified as a gene transcriptionally upregulated by the IIS inhibitor PTEN (Unoki & Nakamura, 2001) (Fig. 1). This transcriptional induction is mediated by Foxo (Mei *et al*., 2009; Sengupta *et al*., 2011). This mitophagy inhibition has important physiological consequences, as mitophagy inhibition prevents the lifespan‐increasing effects of IIS inhibition in nematodes (Palikaras *et al*., 2015). In addition, increasing mitophagy genetically or pharmacologically can extend lifespan in several organisms (Rana *et al*., 2013; Ryu *et al*., 2016).

**Figure 2:**
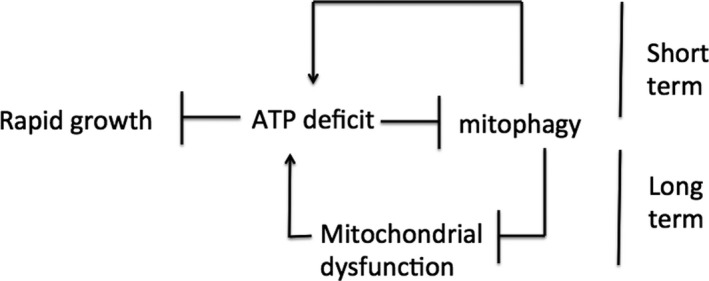
Short‐term and long‐term effects of impaired mitophagy. An ATP deficit impairs mitophagy by activating IIS. This mitophagy impairment prevents premature autophagic destruction of partially functional mitochondria. This impairment increases ATP production and thus facilitates growth in the short term. However, by allowing persistence of damaged mitochondria, this impairment leads to the accumulation of dysfunctional mitochondria and decreased ATP production in the long term. By combining short‐term benefit and long‐term detriment, I suggest that impaired mitophagy underlies antagonistic pleiotropy.

The observation that IIS inhibits autophagy and mitophagy in rapidly growing cells, despite deleterious long‐term consequences, suggests two conclusions. First, that mitochondrial ATP production is limiting, particularly under high growth conditions, suggesting further that rapidly growing cells operate under an ATP deficit. Protein synthesis requires a large expenditure of ATP; the observation that Tor induces mitochondrial protein translation to increase ATP production is consistent with this view (Morita *et al*., 2013). Second, that mitophagy decreases ATP production, at least in the short term; mitophagy requires several hours, and during this time, the engulfed mitochondrion is not able to contribute to ATP production. In this way, overzealous or precocious removal of mostly functional mitochondria will decrease peak mitochondrial ATP production in the short term (Fig. 2).

Experiments performed in invertebrates support both of these conclusions. Activation of mitophagy in nematodes decreases ATP levels in young worms (Ryu *et al*., 2016), and increasing mitophagy by *PINK1* overexpression in the Drosophila eye decreases eye size (Koh *et al*., 2012). Similarly in Drosophila, ubiquitous expression of an activated, but not wild‐type, form of the mitophagy protein Parkin is lethal, and muscle‐specific expression of this activated Parkin decreases muscle function in adults. This result suggests that excessive mitophagy can be deleterious even in adulthood (Shiba‐Fukushima *et al*., 2014). I suggest that as damaged mitochondria accumulate during aging, organisms become increasingly dependent on these mitochondria for necessary ATP production. This increasing dependency, in fact, is what necessitates the decreasing mitophagy during aging. Consistent with this view, the effectiveness of decreased IIS on extending *C. elegans* lifespan progressively diminishes as the decreased IIS is initiated progressively later during aging (Dillin *et al*., 2002). I suggest that the abrupt increase in mitophagy caused by late‐in‐life IIS inhibition leads to a deleterious culling of damaged, but essential mitochondria.

## Mitophagy inhibition as the cellular correlate of antagonistic pleiotropy

An organism that slows its growth through excessive mitophagy will allow out‐competition for scarce nutrients by other organisms. Thus, under rapid growth conditions, cells attain a short‐term selective advantage by inhibiting mitophagy. However, this mitophagy inhibition also allows persistence of mitochondria with damaged DNA, which will eventually lead to decreased mitochondrial ATP production as damaged mitochondria accumulate. Accumulation of damaged mitochondria has been proposed to promote aging (Dutta *et al*., 2012; Palikaras & Tavernarakis, 2012; Carnio *et al*., 2014; Diot *et al*., 2016). Thus, cells attain a long‐term selective disadvantage by inhibiting mitophagy (Fig. 2). The combination of short‐term advantage and long‐term disadvantage suggests that mitophagy inhibition acts as a cellular correlate with AP.

As mitophagy inhibition continues and mitochondrial dysfunction increases, ATP output will decline, exacerbating the ATP deficit. I suggest that as this ATP deficit increases, cells respond by further inhibiting mitophagy in order to salvage higher ATP production. This response eventually leads to a further decrease in mitochondrial ATP production, a further increase in the ATP deficit, and so on, in a classic death spiral (Fig. 2). Ultimately, a catastrophic collapse in ATP production ensues.

In this view, evolution selects for rapid growth as well as slow aging. However, because of the specific biology of mitochondria, organisms cannot simultaneously grow rapidly and age slowly. Organisms will balance these contradictory alternatives to maximize lifetime reproduction. Different species may choose to emphasize either rapid growth or slow aging, and evolutionary niches are available for many different growth/aging strategies. The house mouse combines extremely rapid growth (~20‐day gestation period) with extremely rapid aging (~3‐year lifespan) and presumably low levels of mitophagy, whereas the naked mole rat combines extremely slow growth (~70‐day gestation period) with extremely slow aging (~30‐year lifespan; Roelling *et al*., 2011) and presumably high levels of mitophagy. In addition, organisms are capable of modulating growth rate vs. aging rate upon changes in nutrient availability; this is accomplished by modulating IIS activity and hence mitophagy (Kenyon, 2010). However, the trade‐off between rapid growth and slow aging is never eliminated.

## A proposed mechanism for the mitochondrial death spiral

As mitochondrial output begins to decline during aging, cellular demand for ATP outstrips the ability of mitochondria to produce the required ATP. I suggest that this inadequacy of ATP supply is communicated to the cytoplasm by an increase in mitochondrial ROS production. For example, mouse cardiac cells under metabolic load and Drosophila muscle cells with genetically impaired complex I function increase ROS generation (Sundaresan *et al*., 2009; Owusu‐Ansah *et al*., 2013). Other studies indicate increased ROS generation from mitochondria defective in oxidative phosphorylation (Turrens, 2003; Kregel & Zhang, 2007; Murphy, 2009, 2013; Tal *et al*., 2009; West *et al*., 2011; Raimundo *et al*., 2012). Finally, mitophagy impairment is sufficient to increase ROS generation in yeast (Kurihara *et al*., 2012; Bin‐Umer *et al*., 2014) and human monocytes (Zhou *et al*., 2011). How mitochondrial dysfunction increases ROS generation is not clear. Taken together, these observations indicate that cells respond to initial declines in mitochondrial ATP production by increasing ROS generation, which I suggest signals the cell that mitochondrial ATP output has become inadequate to meet cellular requirements.

I suggest that this ROS increase inhibits mitophagy via IIS activation. ROS has been shown to increase IIS pathway activity at several steps (Fig. 1; Okoh *et al*., 2013; reviewed in Sullivan and Chandel, 2014). First, several intermediates of IIS are activated by ROS either produced endogenously or supplied exogenously. In particular, Ras is activated when the thiol group of cysteine 118 is oxidized (Sawyer *et al*., 2002; Kuster *et al*., 2005; Sundaresan *et al*., 2009). This mechanism might underlie the observation that the activation of Erk by hydrogen peroxide requires Ras activity (Guyton *et al*., 1996). In addition, hydrogen peroxide activates PI3K (Wang *et al*., 2000; Qin & Chock, 2003; Stone & Yang, 2006), and ROS directly activates Tor (Sarbassov & Sabatini, 2005; Reiling & Sabatini, 2006) in part by inducing disulfide bond formation at the C‐terminus, which stabilizes the protein (Dames *et al*., 2005). Second, several IIS inhibitors are themselves inhibited by ROS. PTP1B, a phosphatase that deactivates receptor tyrosine kinases, is inhibited by oxidation, which enables activation of both the insulin and epidermal growth factor receptors (Knebel *et al*., 1996; Denu & Tanner, 1998; Lee *et al*., 1998; Finkel & Holbrook, 2000). In addition, PTEN, which removes the 3’ phosphate from PIP_3_ and thus opposes PI3K activity, is likewise inhibited by ROS (Lee *et al*., 2002; Leslie *et al*., 2003; Connor *et al*., 2005). Finally, ROS inhibits the Tsc1/Tsc2 complex, thereby relieving Tor from upstream inhibition (Yoshida *et al*., 2011). Thus, ROS acts through a variety of targets to activate IIS and Tor. This IIS activation is predicted to inhibit Foxo.

Not only does increased ROS activate IIS, but activated IIS also increases ROS (Irani *et al*., 1997; Trachootham *et al*., 2006; Nogueira *et al*., 2008; Silva *et al*., 2011; reviewed in Dolado & Nebreda, 2008). This increase in ROS likely occurs at least in part via mitophagy inhibition, which as described above is sufficient to activate ROS, although mitophagy‐independent, IIS‐dependent ROS increases might also occur. The ability of IIS and ROS to activate each other supports the notion that a ROS/IIS positive feedback can be generated. Such a positive feedback, once initiated, is anticipated to progressively impair mitophagy, accelerate mitochondrial dysfunction, and irreversibly decrease cellular ATP production.

**Figure 3:**
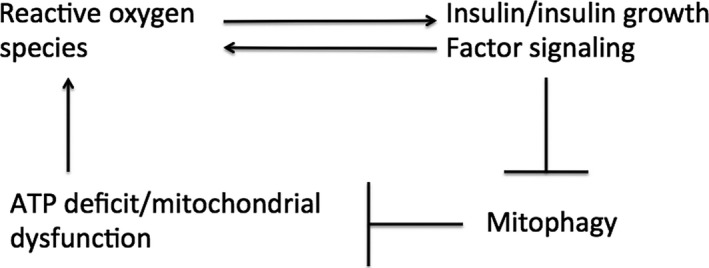
The mitochondrial death spiral. Cellular ATP deficit or mitochondrial dysfunction causes increased production of ROS. This increased ROS activates IIS, which in turn activates ROS by inhibiting mitophagy and thus promoting further mitochondrial dysfunction and exacerbating the cellular ATP deficit. IIS might also increase ROS through mitochondria‐independent mechanisms.

Based on these molecular events, I propose the following model for the mitochondrial death spiral (Fig. 3). As deficit of mitochondrial ATP production continues to rise, the consequent rise in mitochondrial ROS production progressively oxidizes the cytoplasm and increases IIS pathway activity. Mitophagy thus becomes progressively attenuated, further exacerbating mitochondrial decline and thus the ATP supply deficit, in a positive feedback loop.

## Testing predictions of this model

### Mitochondrial dysfunction increases with age

Many lines of evidence indicate that mutations in mitochondrial DNA accumulate and mitochondrial function declines during aging (reviewed in Payne & Chinnery, 2015). Mitochondrial DNA damage increases in aging rodents (Hamilton *et al*., 2001; Genova *et al*., 2004; Hagen *et al*., 2004) and humans (Taylor *et al*., 2003), and these increases in mutation can lead to reduced flow through the electron transport chain during aging (Wanagat *et al*., 2001; Hagen *et al*., 2004; Short *et al*., 2005; reviewed in Golden & Melov, 2001; Ikeda *et al*., 2014).

### Cells become more oxidized with age

The model shown in Fig. 3 suggests that as the mitochondrial death spiral progresses, cells should become progressively more oxidized. This possibility is supported by investigations by several groups (reviewed in Droge, 2003). In particular, older animals generate more oxidation products than younger animals in response to radiation (Beckman & Ames, 1998). In addition, levels of reduced glutathione decline with age both in plasma and in multiple tissues (Maher, 2005; Jones, 2006), perhaps as a consequence of age‐dependent decreases in glucose 6 phosphate dehydrogenase activity (Beckman & Ames, 1998). Finally, it was shown in yeast, nematodes, and Drosophila that the cytoplasm or mitochondria become increasingly oxidized during aging (Liu *et al*., 2012; Brandes *et al*., 2013; Kirstein *et al*., 2015; Knieß & Mayer, 2016). This cellular oxidation might be responsible for the observation that very old Drosophila display strikingly similar changes in gene expression to Drosophila placed under oxidative stress (Landis *et al*., 2004).

### ROS production increases with age

The increase in oxidation state during aging is most likely a consequence, at least in part, of increased generation of ROS from mitochondria. In Drosophila, hydrogen peroxide production significantly increases during aging (Cochemé *et al*., 2011; Sohal & Orr, 2012; Orr *et al*., 2013) and increased ROS release during aging was observed from rodent muscles, heart, liver, and brain (Sohal *et al*., 1994; Bejma & Ji, 1999; Bejma *et al*., 2000; Driver *et al*., 2000; Vasilaki *et al*., 2006; reviewed in Hekimi *et al*., 2011).

### IIS becomes more activated with age

Several lines of evidence suggest that Foxo activity diminishes during aging in the rat muscle and kidney (Edström *et al*., 2006; Kim *et al*., 2008, 2014). In addition, the transcription of several autophagy genes decreases during aging in the Drosophila flight muscle. This decrease is mostly likely due to decreased Foxo activity, as ectopic overexpression of *Foxo* is sufficient to rescue this transcriptional decrease (Demontis & Perrimon, 2010). Finally, transcription of the Foxo‐dependent mitophagy gene *PINK1* is downregulated during aging in the mouse lung (Sosulski *et al*., 2015).

The effect of aging on Tor activity is less clear. Although Tor activity was reported to increase with age in muscle, liver, lung, and stem cells (Chen *et al*., 2009; Sandri *et al*., 2013; Leontieva *et al*., 2014; reviewed by Nacarelli *et al*., 2015; Romero *et al*., 2016; White *et al*., 2016), other studies failed to confirm some of these findings (Baar *et al*., 2016). It appears that phosphorylation of various Tor substrates is affected differentially during aging. Unfortunately, phosphorylation status of autophagy components is difficult to evaluate due to lack of phospho‐specific antibodies. In addition, Tor activity is less effective in regulating autophagy when Foxo activity is low (Mammucari *et al*., 2007), most likely because of the low expression of autophagy proteins.

### Autophagy declines with age

Many lines of evidence demonstrate that autophagy declines with aging (reviewed in Keller *et al*., 2004; Bergamini *et al*., 2004; Massey *et al*., 2006; Cuervo, 2008; Rubinsztein *et al*., 2011; Kroemer, 2015; Romero *et al*., 2016). Autophagy declines during normal aging in Drosophila muscle (Demontis & Perrimon, 2010), rat liver (Del Roso *et al*., 2003); mouse lung (Shirakabe *et al*., 2016), and human brain (Keller *et al*., 2004; Lipinski *et al*., 2010). Finally, the mitophagy inducer *PINK1* is transcriptionally downregulated during aging in mouse lung (Sosulski *et al*., 2015).

The declines in mitophagy during aging might be causally related to declines in mitochondrial biogenesis also observed during aging (Vina *et al*., 2009; Seo *et al*., 2010). Declines in mitochondrial biogenesis are likely caused in part by decreased levels of transcription factors such as PGC‐1α and NRF1 that increase expression of mitochondrial genes (Baker *et al*., 2006; Finley & Haigis, 2009). In addition, PGC‐1α activity is inhibited by Akt‐dependent phosphorylation (Li *et al*., 2007), which might link the observed increase in IIS during aging with attenuated mitochondrial biogenesis. Thus, cells combine attenuated mitophagy with attenuated mitochondrial biogenesis, which enables total mitochondrial mass to be held within controlled limits.

## Greatly elevated cytoplasmic oxidation late in life: implications for oxidative stress, mitohormesis, and insulin resistance

Different levels of ROS confer distinct cellular effects. Low levels of ROS induce growth, protein synthesis, and proliferation (Antunes & Cadenas, 2001; Kwon *et al*., 2003; Cadenas, 2004). I suggest that these ROS levels are generated as the mitochondrial death spiral progresses. In contrast, higher ROS levels can induce an oxidative stress response that involves JNK activation and Tor inhibition (Reiling & Sabatini, 2006; Takimoto and Kass, 2007). Activated JNK, in turn, activates Foxo by phosphorylation, which overcomes the Foxo nuclear import barrier induced by IIS (Oh *et al*., 2005; Tzivion *et al*., 2011) and enables Foxo activity despite Akt‐dependent phosphorylation (Wang *et al*., 2005). This Foxo activation is necessary for activated JNK to increase lifespan (Wang *et al*., 2005). Although the mechanism underlying the Tor inhibition that occurs under high ROS is not completely clear, it is possible that a role is played by the ROS‐dependent activation of AMPK (Cardaci *et al*., 2012), which inhibits Tor both by phosphorylating and activating the Tor inhibitor Tsc1/Tsc2 (Fig. 1; Inoki *et al*., 2003) and by phosphorylating and inhibiting the Tor‐associated scaffold Raptor (Gwinn *et al*., 2008). JNK is activated during aging in the rodent brain and liver and in the gut of very old Drosophila (Suh, 2001; Hsieh *et al*., 2003; Williamson *et al*., 2003; Biteau *et al*., 2008; Zhou *et al*., 2009); this gut JNK activation is at least partly responsible for the Foxo activation that occurs in these very old Drosophila (Guo *et al*., 2014). Taken together, these results raise the possibility that very late in life, the cytoplasm can become oxidized sufficiently to induce an oxidative stress response that reactivates Foxo and inhibits Tor. These late‐stage effects on Foxo and Tor are predicted to induce a cellular switch to mitochondrial protection late in life. Mitochondrial protection triggered by oxidative stress is termed ‘mitohormesis’ (Tapia, 2006).

I suggest that induction of mitohormesis by high ROS production explains at least in part the well‐established observation that pharmacologically or genetically crippling ATP production is capable of increasing lifespan (Lee *et al*., 2003; Schulz *et al*., 2007; Copeland *et al*., 2009; Owusu‐Ansah *et al*., 2013; Sun *et al*., 2014). For example, Owusu‐Ansah *et al*. (2013) reported that the increased lifespan caused by knockdown of the complex I subunit ND75 is accompanied by, and requires, a ROS increase, followed by JNK activation, transcriptional induction of several Foxo target genes, including *4E‐BP*,* InR,* and *ImpL2*, and increased mitophagy (Owusu‐Ansah *et al*., 2013). In addition, Schulz *et al*. (2007) reported that impaired glycolysis extended lifespan by the ROS‐dependent activation of AMPK (Schulz *et al*., 2007). In a similar manner, feeding superoxide generators can increase lifespan in *C. elegans* (Yang & Hekimi, 2010). This lifespan increase requires Foxo (Heidler *et al*., 2010) and thus might be due to JNK‐dependent mitohormesis as well. Taken together, these results indicate that high ROS levels, beginning early in life, enable cells to bypass the mitochondrial death spiral and proceed directly to the late‐stage mitohormetic state, and that this phenomenon is responsible for the increased lifespan observed.

Furthermore, cytoplasmic oxidation sufficient to promote an oxidative stress response might also be relevant to understanding the insulin resistance (IR) that often develops in the elderly. Oxidative stress and JNK are implicated in IR (Salmon, 2012); JNK phosphorylates the insulin receptor substrate 1 (IRS‐1) and attenuates the ability of ligand‐bound insulin receptor to activate IRS‐1 (Aguirre *et al*., 2000). A JNK deletion at least partly restores insulin sensitivity in a mouse obesity model (Hirosumi *et al*., 2002), indicating that this JNK‐dependent phosphorylation is functionally relevant. Given the observation that oxidative stress is increased during aging (Mendoza‐Núñez *et al*., 2011), these results suggest that age‐dependent IR, like the late‐stage mitohormetic state, occurs at least in part when the mitochondrial death spiral‐induced cytoplasmic oxidation progresses sufficiently to activate JNK.

Increased ROS also promotes mitochondrial protection through activation of the transcription factor Nrf2, which triggers expression of a number of antioxidant genes (Ristow & Schmeisser, 2014). Increased expression of antioxidants is expected to attenuate the ROS‐mediated positive feedback (Fig. 3), and the observation that Nrf2 activity shows dose‐dependent effects on lifespan (An *et al*., 2005) is consistent with this attenuation. However, Nrf2 activity declines with age (Suh *et al*., 2004), which likely occurs at least in part by increased Tor‐dependent inhibition of Nrf2 activity (Robida‐Stubbs *et al*., 2012; Lerner *et al*., 2013). Loss of Nrf2 activity with age will thus weaken the ability of Nrf2 to attenuate the positive feedback and might play a part in permitting the positive feedback shown in Fig. 3 to accelerate during aging.

## Role of antioxidants in lifespan

The hypothesis proposed here predicts that antioxidant administration, if applied before the mitohormetic state develops, should extend lifespan. However, data on the effects of antioxidant administration have been difficult to interpret. Although ectopic overexpression of the peroxiredoxin *Prx5* increases lifespan in Drosophila (Radyuk *et al*., 2009), indicating that decreasing ROS production can attenuate the mitochondrial death spiral as expected, the effects of feeding antioxidants on lifespan have been inconsistent. Difficulties in enabling antioxidant access to the cytoplasm might represent one issue, as the effectiveness of specific antioxidants can vary depending on the precise method of antioxidant presentation (Shibamura *et al*., 2009; in data interpretation include the difficulty in determining the Desjardins *et al*., 2017). Additional difficulties extent to which the antioxidant feeding actually reduces the cytoplasm. Fluorescent ROS indicators that monitor light production from the whole organism are problematic as these combine signal from the various subcellular and extracellular compartments, including the cytoplasm, mitochondria, peroxisomes, ER, and extracellular space, which all possess different redox states. This complicates ability to isolate redox changes specific to the cytoplasm. In addition, it is often difficult to distinguish lifespan effects due to antioxidant properties from beneficial or toxic effects of the compounds distinct from antioxidant properties. From these results, I suggest that the ability of antioxidants to increase lifespan remains an unresolved question.

## Future work and limitations and extensions of this model

Causality has not yet been determined for several proposed events. Thus, for example, it is not yet known whether the increased oxidation state of the aging cytoplasm is causal for increased IIS. Further studies will be needed to address this issue. Second, this analysis deals specifically with only one mechanism proposed to underlie AP. It is likely that AP is also driven by other mechanisms. In addition, it is also likely that processes independent of AP drive aging. Such non‐AP aging processes could arise as a consequence of decreased selective pressure in old age. Finally, the ROS‐IIS positive feedback system described here is likely to advance aging through processes in addition to loss of mitochondrial ATP production. For example, the age‐dependent activation of Tor and loss of Foxo described above are predicted to inhibit autophagy in general. This progressive loss of autophagy, combined with increased ROS and thus ROS‐mediated oxidative damage, might be responsible in part for the loss of proteostasis that occurs during aging and likely plays a critical role in the aging process. This loss of proteostasis is manifested by the accumulation of protein aggregates, inclusion bodies, and other damaged macromolecules, which are degraded via autophagy (Yao, 2010). The possibility that Tor activation during aging might be partly responsible for the accumulation of these damaged macromolecules has led to the suggestion that rapamycin administration might be helpful in reversing this accumulation. In addition, Foxo plays a critical role in inducing expression of components of the proteasome as well as components of the autophagosome (Webb & Brunet, 2014). Thus, loss of Foxo activity during aging is likely to contribute to loss of proteostasis through multiple outputs.

## You cannot get there from here

The mitochondrial death spiral should not be viewed as an error in cell physiology. Rather, this death spiral should be viewed as a deliberate, sensible, in fact necessary, approach by cells to solve the difficult problem of successfully growing and reproducing in a competitive world of scarce nutrients. This problem is exacerbated by the fact that cells have not yet been able to relocate every mitochondrial gene to the nucleus. This deficit means that the simultaneous demand for both high ATP production and high mitochondrial quality control becomes contradictory. The mitochondrial death spiral represents a cellular attempt to resolve these contradictory demands.

It might seem plausible to dispose of the mitochondrial death spiral after growth and reproduction are complete. Thus, when very high levels of ATP production are no longer needed, perhaps an aging cell could rejuvenate by greatly amplifying the few pristine mitochondria that might remain and degrading the rest. However, in practice, this course of action might require as a temporary intermediate a lethal decline in ATP production. Extrication from a death spiral once initiated is not easy. If a path from one point to another requires transit through a lethal state, then this path cannot be taken, regardless of the attractiveness of the destination (see John Maynard Keynes’ quote above). Evolution has not found a way to overcome this drawback. That is not to say that this problem is intractable. Perhaps this problem could be solved by organisms possibly more ingenious and certainly more motivated than evolution.

## Funding

No funding information provided.

## Conflict of interest

I have no conflicts of interest to declare.
